# Longitudinal Associations of Experiential and Reflective Dimensions of Meaning in Life With Psychopathological Symptoms

**DOI:** 10.32872/cpe.11381

**Published:** 2024-09-30

**Authors:** Albert Anoschin, Michael K. Zürn, Carina Remmers

**Affiliations:** 1 Department of Psychology, Institute for Mental Health and Behavioral Medicine, HMU Health and Medical University Potsdam, Potsdam, Germany; 2 Nuremberg Institute for Market Decisions (NIM), Nuremberg, Germany; Friedrich-Alexander-Universität Erlangen-Nürnberg, Erlangen, Germany

**Keywords:** meaning in life, reflection, depression, personality functioning, longitudinal study

## Abstract

**Background:**

Rather than being rooted in deliberate reflection, the experience of meaning has been shown to evolve from intuitive processes (Heintzelman & King, 2013b, https://doi.org/10.1007/978-94-007-6527-6_7). Accordingly, experiential and reflective dimensions of meaning in life can be distinguished (Hill et al., 2019, https://doi.org/10.1080/09515070.2018.1434483). In this preregistered study, we explored how these dimensions are longitudinally associated with psychopathological symptoms. We expected that experiencing more meaning would predict fewer depressive symptoms and fewer personality functioning impairments six months later, whereas reflecting about meaning would predict more psychopathological symptoms.

**Method:**

A German-speaking sample of *N* = 388 completed self-report measures assessing meaning in life, depression, and personality functioning at baseline and six months later.

**Results:**

Controlling for depression at baseline, elevated levels of experiencing meaning in life predicted a decrease in depressive symptoms. Experiencing meaning did not predict personality functioning impairments six months later. However, exploratory analyses with a larger sample tentatively showed that experiencing meaning in life predicted less impairments in personality functioning. Evidence supporting the hypothesized association between reflection and future depression as well as future personality functioning impairments was discerned through exploratory analyses. Generalizability of results to clinical care settings is limited due to the studied non-clinical sample. No causal conclusions can be drawn from the data because the study employed an observational design with two assessment points.

**Conclusion:**

Experiencing meaning in life emerged as a potential protective factor against future psychopathological symptoms, whereas exploratory analyses pointed to an opposite relationship for reflection about meaning in life. Results are discussed with regard to clinical implications and directions for future research.

## Highlights

- Increased experience of meaning in life may contribute to a reduction in depressive symptoms and personality functioning impairments.
- Preliminary evidence suggests that reflection about meaning is associated with future personality functioning impairments and depressive symptoms.
- Further investigation of bi-directional relationships between meaning in life and psychopathology is suggested.

Experiencing one’s life as meaningful is associated with adaptive coping (Miao et al., 2017; Ward et al., 2023), physical health (Czekierda et al., 2017; Hooker et al., 2018) and various indicators of psychosocial well-being. These include self-esteem, goal attainment, satisfaction with life and satisfaction with interpersonal relationships (J.-B. Li et al., 2021; Morgan & Robinson, 2013; Schultheiss, 2021; Soucase et al., 2023). Conversely, existential meaninglessness goes along with adverse mental health outcomes, such as depression, suicidal ideation and addictive behaviors (Hu et al., 2022; W. Li et al., 2020; Schnell et al., 2018). Are existential crises merely by-products of depression, or is meaning in life a relevant marker that predicts the extent to which a person will suffer from depression in the future? Despite its clinical relevance, only few studies have investigated this question longitudinally (Dulaney et al., 2018; Krause, 2007; Mascaro & Rosen, 2008; Park et al., 2020). For instance, Mascaro and Rosen (2008) performed a cross-lagged panel analysis with *N* = 395 students and found that higher levels of meaning were significantly associated with less depressive symptoms two months later. Although the authors used multiple instruments to assess meaning in life, they combined these measurements into one ‘global sense of meaning’ factor. A recent account by Hill et al. (2019), however, suggests that a core distinction between the reflective and experiential dimension of meaning in life should be made. Cross-sectional findings indicate that these dimensions are differentially related to psychopathology (Remmers et al., 2023). With the current study, we aimed to investigate the presumably differential contributions of experiencing meaning and reflecting about meaning to future psychological impairment.

## Experiencing and Reflecting on Meaning and the Association With Psychopathology

Research on meaning in life has so far mostly dealt with the question how people *construct* meaning (for an overview see Park, 2010). This line of research emphasizes that meaning in life is something people must establish actively. However, the subjective sense of life being meaningful may be experienced in the absence of deliberate meaning making (Heintzelman & King, 2013b). Hill et al. (2019) proposed a framework with two distinct meaning in life dimensions termed *experience* and *reflection,* fitting with the idea that experiencing meaning can be dissociated from cognitive occupation with meaning. This conceptualization is in line with dual-process theories of cognition (Thompson et al., 2011) that differentiate experiential and affective processing modes from abstract, reflective processing modes. Clinical accounts point to a differential adaptivity of these processing modes when faced with stressful experiences (Watkins et al., 2008). Prior research has shown that the experience of meaning arises from an intuitive process which enables persons to recognize coherent patterns in their experiences and environments (Heintzelman & King, 2013a). This intuitive process operates unconsciously, fast and associatively. As a result, intuitions of coherence and meaning are phenomenologically experienced as “knowing something without knowing how one knows” (Bowers et al., 1990; Topolinski & Strack, 2009). Given the fast and easy nature of the underlying process, detecting coherence automatically feels right (Thompson et al., 2011) and positive (Topolinski & Strack, 2008). When no meaning is found intuitively, a deliberate reflective process may follow (Thompson et al., 2011). Ironically, trying to find rational explanations why life has meaning may neither be conducive to psychological functioning nor to perceiving meaning on an experiential level (J.-B. Li et al., 2021; Topolinski & Strack, 2008). Several studies show that people who report searching for meaning also experience being more depressed, less happy and less satisfied with life (Soucase et al., 2023; Steger et al., 2006, 2009). In a similar fashion, reflective thought about meaning may be maladaptive in some individuals (Watkins & Roberts, 2020), because it overrides intuitive meaning cues and disrupts the subjective experience of meaning (Remmers et al., 2023; Topolinski & Strack, 2008).

## The Current Study

We aimed to determine whether experience of meaning in life and reflection on meaning in life prospectively predict depressive symptoms over a time-period of six months. In a recent cross-sectional study, experiencing meaning was negatively associated with depression, whereas reflecting on meaning showed a positive association with depression (Remmers et al., 2023). The aim of the current study was to elucidate such differential relationships longitudinally. If experiencing meaning turns out to be prognostically favorable for mental health, this would tentatively suggest that practitioners should focus on the experiential level when designing interventions. If, at the same time, a positive longitudinal association between reflection and depression were found, this would support the view that increased cognitive preoccupation with topics of meaning in life may be detrimental to mental health.

We expected that the hypothesized associations would not be specific to depression but generalize to personality functioning impairments as a global indicator of psychopathology. Personality functioning refers to a person’s ability to regulate the self and interpersonal relationships. Impairments in self-other regulation are to be expected in persons with various psychopathological symptoms (Friborg et al., 2014). The construct of personality functioning impairments accommodates recent dimensional conceptualizations of psychopathology (Caspi & Moffitt, 2018; Forbes et al., 2021) and lies at the core of the diagnostic approach to personality pathology in the ICD-11 and the Alternative DSM-5 Model for Personality Disorders (Bach et al., 2020; Bach & Simonsen, 2021). Some authors propose that personality functioning represents the p-factor explaining a general vulnerability for psychopathology (Bender, 2019).

## Hypotheses

In our preregistered hypotheses, we expected more experience of meaning in life at baseline (T1) to predict less depression and less impairments in personality functioning six months later (T2). Conversely, we expected that participants’ propensity to reflect about meaning in life at T1 would be predictive of more depressive symptoms and greater personality functioning impairments at T2. Importantly, in all our analyses we controlled for baseline levels of psychopathological symptoms, as we were interested in the unique predictive value of experiential and reflective dimensions of meaning in life. The study design was preregistered prior to follow-up data collection^1^1Note that additional hypotheses regarding the predictive role of intuitive coherence detection for meaning in life and psychopathology were preregistered. As these associations were not of interest for the current thrust, we opted not to report them in the present article. (Remmers et al., 2022S).

## Method

### Sample

Participants were recruited via Prolific (www.prolific.co), an online participant pool for academic research. All measures were administered online. Participants provided informed consent and were compensated for participation. The follow-up study was displayed on the Prolific website only to persons who participated in the baseline assessment (*N* = 1,189; see Remmers et al., 2023). These participants could choose to participate at follow-up voluntarily, but were not actively contacted or requested to participate. From the baseline sample, *n* = 538 participants completed the follow-up assessment (response rate: 45%).

In our preregistration we stated that analyses should only include participants who took part within the first two weeks of the baseline assessment. We hereby aimed to keep the time interval constant between baseline and follow-up. Confirmatory hypothesis tests reported below pertain to this preregistered sample, which consisted of *n* = 388 participants. Within this analyzed sample (Age: *M* = 27, *SD* = 9), 69% identified as female, 29% identified as male and 2% identified as non-binary. 33% of the participants had a university degree, 6% had an advanced technical certificate, 52% had a high-school degree, 7% had a secondary school degree and 1% had a lower secondary school degree.

Since the procedure to only include participants from the first two weeks of the baseline assessment resulted in a loss of statistical power, we decided to relax our preregistered restriction in a second step and conducted further analyses with the total follow-up sample (*n* = 538). Within the total sample, the median interval between baseline and follow-up assessment was 202 days (range: 164 to 238 days), which corresponds to approximately 6.66 months. The higher-powered analyses with the full sample are reported in the Exploratory Analyses section.

### Procedure

The study was part of a larger research project which was approved by the ethics committee of the Freie Universität Berlin (proposal number 005/2019).

#### Baseline (T1)

At baseline, participants filled out questionnaires in randomized order, assessing meaning in life, depression and personality functioning impairments. Additional self-report instruments and a behavioral task were employed, which were not relevant for the current study and are detailed in Remmers et al. (2023). Participants were reimbursed immediately after completing the baseline assessment.

#### Follow-up (T2)

The follow-up assessment was made available six months later for participants who completed the baseline assessment. The same self-report instruments that assessed the meaning in life dimensions and psychopathology were re-applied.

### Measures

#### Meaning in Life

Experience of meaning in life and reflection on meaning in life were assessed with the German translation (Anoschin et al., 2022) of the Meaning in Life Measure (Hill et al., 2019). Participants rated their agreement to eight items on a 9-point Likert scale (exemplary experience item: “I experience my life as meaningful”; exemplary reflection item: “I think about what gives me meaning”). Verbal markers were placed at the scale points 1 (“strongly disagree”), 5 (“neutral”) and 9 (“strongly agree”). We computed McDonald’s ω as reliability estimate, yielding ω = .75 for the experience scale and ω = .85 for the reflection scale.

#### Depressive Symptoms

The German version of the 8-item Patient Health Questionnaire was used to assess presence of depressive symptoms at baseline and follow-up (PHQ-8; Kroenke et al., 2009). Participants rated items on a 4-point Likert scale that queried depressive symptoms in the past two weeks (e.g., “feeling tired or having little energy”). When used as a screening instrument, a cutoff point ≥ 10 can be applied to detect current major depression with high diagnostic accuracy (Wu et al., 2020). Reliability of the PHQ-8 in the present study was ω = 86.

#### Personality Functioning Impairments

We used the Level of Personality Functioning Scale Brief Form (LPFS-BF; Spitzer et al., 2021) to assess impairments in personality functioning. Participants rated 12 items on a 4-point Likert scale (e.g., “I often think very badly of myself”). The measure showed a reliability of ω = .88.

### Systematic Dropout

To explore systematic dropout, we investigated whether and with respect to which variables participants (from the first two weeks of the baseline assessment) who participated in the follow-up assessment differed from participants (from the two first weeks of baseline assessment) not participating in the follow-up assessment. We found that participants who participated at T2 were significantly older, *t*(767.39) = 3.85, *p* < .001; *M*_Δ_ = 2 years, reported less depressive symptoms, *t*(797.73) = -2.27, *p* = .024; *M*_Δ_ = -0.9, and less impairments in personality functioning, *t*(788.46) = -2.48, *p* = .013, *M*_Δ_ = -1.16. Age and psychopathological symptoms were significantly correlated, with older participants reporting a lower burden (*r* = -.22, *p* < .001 for PHQ-8; *r* = -.24, *p* < .001 for LPFS-BF). Due to this data pattern, and conforming to our preregistration, we conducted regression analyses with a full information maximum likelihood approach (FIML), including age as an auxiliary variable (Enders, 2008; Graham, 2003).

## Results

Descriptive statistics and zero-order correlations for the self-report measures across both time-points are summarized in Table 1 and Table 2, respectively. Descriptive statistics for the full sample are listed in Anoschin et al., 2024S, Table S1. Experience of meaning in life scores (*M*_t1_ = 6.05, *M*_t2_ = 6.03) were descriptively above the scale midpoint of 5, but lower than scores observed by Hill et al. (2019) in two US samples (*M*_Study1_ = 7.45, *M*_Study2_ = 7.13). Reflection scores (*M*_t1_ = 6.50, *M*_t2_ = 6.19) were within a similar range to those observed by Hill et al. (*M*_Study1_ = 6.52, *M*_Study2_ = 6.74). We noted a substantial prevalence of depressive symptoms in our studied sample. When applying the recommended PHQ-8 cutoff ≥ 10 (Kroenke et al., 2009), 48% of participants screened positive for major depression at baseline, and 43% at follow-up. Scores of the LPFS-BF corresponded to norm values of *T* = 64 at baseline and *T* = 63 at follow-up, indicating above-average impairments in personality functioning within our sample (Spitzer et al., 2021).

**Table 1:** Descriptive Statistics of Meaning in Life and Psychopathology Measures (N = 388)

Measures	Baseline	Follow-Up
	*M* (*SD*)	*M* (*SD*)
Meaning in Life		
Experience	6.05 (1.45)	6.03 (1.39)
Reflection	6.50 (1.57)	6.19* (1.69)
**Depression**	9.78 (5.51)	9.02* (4.79)
**Personality Functioning**	27.15 (6.77)	25.60* (6.90)

*Note.* Asterisks indicate significant differences in scores between baseline and follow-up assessments (based on paired *t*-tests, *p* < .001). Meaning in Life: scale means of MILM (possible range: 1 – 9); Depression: scale sums of PHQ-8 (possible range: 0 – 24); Personality Functioning: scale sums of LPFS-BF (possible range: 12 – 48, higher scores reflect greater impairment).

**Table 2:** Bivariate Correlations Among Study Variables for Both Assessment Time Points (N = 388)

Measures	1	2	3	4	5	6	7	8
1. T1 MIL Experience	–	0.19***	-0.48***	-0.58***	0.69***	0.12*	-0.40***	-0.49***
2. T1 MIL Reflection		–	0.18***	0.13*	0.10	0.63***	0.15**	0.15**
3. T1 Depression			–	0.69***	-0.44***	0.12*	0.68***	0.61***
4. T1 Personality Functioning				–	-0.50***	0.12*	0.58***	0.79***
5. T2 MIL Experience					–	0.17***	-0.47***	-0.52***
6. T2 MIL Reflection						–	0.10*	0.14**
7. T2 Depression							–	0.66***
8. T2 Personality Functioning								–

*Note*. MIL: Meaning in Life (MILM; Hill et al., 2019); Depression: PHQ-8 (Kroenke et al., 2009); Personality Functioning: LPFS-BF (Spitzer et al., 2021).

*p < .05. **p < .01. ***p < .001.

### Confirmatory Hypotheses Testing

We preregistered OLS regression analyses with two-sided significance tests^2^2We did not conduct an a priori power analysis because the achievable sample size was limited by the number of persons who participated in the baseline assessment. A post-hoc sensitivity analysis showed that, given our sample size and an alpha error probability of α = .05, an effect size of *f*^2^ = .02 would be required to be detectable with a power of .80. (Remmers et al., 2022S). In each model, the dependent variable at T2 was regressed on the independent variable at T1, controlling for effects of the dependent variable at T1. As hypothesized, lower levels of experienced meaning in life at T1 significantly predicted more depressive symptoms at T2 (six months later), controlling for depressive symptoms at T1. In contrast, experienced meaning in life at T1 did not significantly predict impairments in personality functioning at T2. We further hypothesized that higher levels of reflection on meaning in life would be associated with more psychopathological symptoms six months later. However, reflection did not significantly predict future depression nor future personality functioning impairments when statistically controlling for psychopathology at T1. The results of the regression analyses are summarized in Table 3 and Table 4.

**Table 3:** Regression Analyses Predicting Depressive Symptoms at T2 From Experienced Meaning in Life and Reflection on Meaning in Life Assessed Six Months Prior, at T1

Measures	T2 Depression							
	Model 1				Model 2			
	β	95% CI	*p*	*f^2^*	β	95% CI	*p*	*f^2^*
T1 Depression	.641	[.574, .708]	< .001	0.601	.680	[.627, .732]	< .001	0.846
T1 MIL Experience	-.093	[-.177, -.009]	.031	0.014				
T1 MIL Reflection					.033	[-.043, .109]	.389	0.002
*R* ^2^	.477				.471			

*Note.* Regression analyses are based on observed variables and were computed with the full-information maximum likelihood method using a sample of *N* = 800 at T1 and *N* = 388 at T2. Age was entered as an auxiliary variable. *p* statistics are based on two-tailed tests as preregistered. MIL: Meaning in Life (MILM; Hill et al., 2019); Depression: PHQ-8 (Kroenke et al., 2009).

**Table 4:** Regression Analyses Predicting Personality Functioning Impairments at T2 From Experienced Meaning in Life and Reflection on Meaning in Life Assessed Six Months Prior, at T1

Measures	T2 Personality Functioning							
	Model 1				Model 2			
	β	95% CI	*p*	*f^2^*	β	95% CI	*p*	*f^2^*
T1 Pers. Func.	.761	[.704, .818]	< .001	0.960	.778	[.742, .814]	< .001	1.551
T1 MIL Experience	-.042	[-.119, .036]	.295	0.003				
T1 MIL Reflection					.053	[-.011, .116]	.105	0.007
*R* ^2^	.616				.617			

*Note.* Regression analyses are based on observed variables and were computed with the full-information maximum likelihood method using a sample of *N* = 800 at T1 and *N* = 388 at T2. Age was entered as an auxiliary variable. *p* statistics are based on two-tailed tests as preregistered. MIL: Meaning in Life (MILM; Hill et al., 2019); Pers. Func.: Personality Functioning Impairments (LPFS-BF; Spitzer et al., 2021).

### Non-Preregistered Exploratory Analyses

For exploratory purposes, we reconducted our analyses including experienced meaning in life and reflection on meaning in life simultaneously as predictors into one regression model. As we aimed to increase power, regression models were computed with the total follow up-sample of *n_T2_* = 538. Given this sample size, it would be possible to detect an effect of *f*^2^ = .014 with a power of .80. This procedure resulted in two linear regression models, one with depression at T2 and one with personality functioning T2 as dependent variables. Again, T1 levels of psychopathology were entered as control variable and age was entered as auxiliary variable using the FIML approach. Exploratory analyses confirmed the negative and significant association of experiencing meaning in life at T1 with depressive symptoms at T2 (β = -.133, 95% CI [-0.205, -0.061], *p* < .001, *f*^2^ = 0.023), when controlling for reflection and depressive symptoms at T1. In contrast to our preregistered analyses but in line with our hypothesis, experiencing meaning in life at T1 significantly predicted less personality functioning impairments at T2 (β = -.085, 95% CI [-0.156, -0.014], *p* = .019, *f*^2^ = 0.010), controlled for reflection and personality functioning at T1. Furthermore, deviating from the results of the preregistered analysis but in line with our hypothesis, reflection about meaning in life at T1 was now significantly and positively associated with depression at T2 (β = .085, 95% CI [0.021, 0.149], *p* = .009, *f*^2^ = 0.010), and with personality functioning impairments at T2 (β = .072, 95% CI [0.014, 0.131], *p* = .016, *f*^2^ = 0.010). Regression tables for these analyses are presented in Anoschin et al., 2024S (Table S2 and Table S3).

In a further step, we explored bidirectional effects between experienced meaning in life, reflection on meaning in life and psychopathology. For this purpose, we fitted two separate cross-lagged structural equation models (SEM) that included meaning in life experience and reflection at T2 as dependent variables. Effects of meaning in life at T1 on psychopathology at T2 were significant and consistent with the exploratory analyses reported above. Additionally, the cross-lagged models indicated significant negative associations between depression at T1 and experience of meaning in life at T2 (β = -.151, 95% CI [-0.223, -0.078], *p* < .001). Depression at T1 was not significantly associated with reflection about meaning in life at T2 (*p* = .523). Similarly, personality functioning impairments at T1 predicted lower experienced meaning in life at T2 (β = -.178, 95% CI [-0.252, -0.103], *p* < .001), but were not significantly associated with reflection about meaning in life at T2 (*p* = .085). SEMs are illustrated in Anoschin et al., 2024S (Figure S1 and Figure S2).

## Discussion

In this preregistered longitudinal study, we examined whether experiential and reflective dimensions of meaning in life would uniquely predict psychopathology six months later in a general population sample. Participants in our online sample presented with varying levels of depression severity and impairments in personality functioning. As hypothesized, we found that participants who experienced more meaning in life reported fewer depressive symptoms six months later, even after controlling for baseline levels of depression. This observation underscores the potential clinical relevance of meaning in life. It is consistent with research suggesting that a diminished experience of life being meaningful may act as a risk factor for the onset or worsening of depressive symptoms (Glaw et al., 2017; Steger, 2022). Conversely, our data suggests that fostering the experience of meaning in life, even in the presence of depressive symptoms, may aid in symptom reduction over time.

Assuming that the benefit of experiencing meaning in life extends beyond depressive symptomatology, we hypothesized that a heightened experience of meaning would predict fewer subsequent impairments in personality functioning. Our preregistered analyses did not support this hypothesis. However, when relaxing our preregistered constraints and conducting our analyses in a larger sample, we discovered a significant negative association between experienced meaning in life and future personality functioning impairments, aligning with our hypothesis. Should this finding be reproducible and robust, it would emphasize that undergoing an existential crisis could not only pose a risk for developing depressive symptoms but may ultimately result in disturbances of self and interpersonal functioning.

It must be noted that in regression analyses, symptom severity at baseline explained a large proportion of variance in symptom severity at follow-up. In comparison, the unique predictive effects of meaning in life on future psychopathology were very small. We suppose that this pattern of results is attributable to conceptual and statistical overlaps between the studied constructs. For example, it is conceivable that core symptoms of depression, such as feeling hopeless, are strongly negatively associated with aspects of meaning in life, such as having a goal in life. Hence, the true contribution of meaning in life to the progression of psychopathological symptoms may be underestimated when both variables are entered simultaneously into a regression model. We advise to take into account the substantial zero-order correlations when assessing the clinical relevance of meaning in life for predicting future mental health outcomes (see Table 2).

In the current study, we focused on potential benefits and drawbacks of meaning in life within the context of prospective psychological impairments. Contrasting with cross-lagged findings reported by Mascaro and Rosen (2008), we also found preliminary evidence for bidirectional effects. Personality functioning impairments and depression at baseline were associated with less experienced meaning at follow-up. Although this does not confirm a causal relationship, it is conceivable that greater interpersonal and self-regulatory ability may strengthen the experience of meaning in life. Potential mediators of this effect may be greater positive mood and better satisfaction of basic psychological needs, such as the need for social relatedness (Autin et al., 2022; Demirbaş-Çelik & Keklik, 2019; Martela et al., 2018). The absence of such experiences that occurs in psychopathology is likely to unfold negative prospective effects on the experience of meaning in life. Conversely, the experience of meaning in life seems to promote positive mood and adequate self-regulation, thereby protecting against psychopathology (Dulaney et al., 2018; Miao et al., 2017). Future studies should explore in more detail the directional dynamics between self- and interpersonal functioning, need satisfaction, and the experience of meaning. Daily diary and experience sampling designs are promising approaches for such endeavor (Kaurin et al., 2023).

### Is Reflection About Meaning Harmful?

Both at baseline and follow-up, more psychopathological symptoms were associated with heightened reflection about meaning in life. This cross-sectional finding suggests that a “low experience, high reflection” pattern might evolve as a trans-diagnostic marker for psychopathology such as depression and impaired personality functioning (see also Remmers et al., 2023). This raises the question about prospective effects of reflection. The literature suggests that persons suffering from depression lack experiential sources of meaning, such as fulfilling social contacts, daily routines or elaborated life goals (King & Hicks, 2021). This condition may lead them to be more preoccupied with the topic of meaning in life (Cohen & Cairns, 2012). However, it is unclear whether reflection about meaning contributes to depressive symptoms. Although reflection did not emerge as significant predictor in our preregistered analyses, exploratory analyses in a larger sample provide preliminary evidence for a positive link between reflection about meaning in life and future psychopathology.

Ultimately, *how* one reflects about meaning in life may be more important than *how much* one reflects about it. For example, it is conceivable that abstract ruminative thinking about meaning in life exacerbates depressive symptoms (Watkins & Roberts, 2020), and such maladaptive cognitive schemas are more likely to be found in persons who already suffer from depression. Future studies should therefore explore how reflection about meaning interacts with self-regulatory success when symptom severity, cognitive schemas and maladaptive personality traits are taken into account (Kerber et al., 2022). In contrast, reflecting upon practical sources of meaning in daily life may be beneficial for attending to these sources in the future (Takano & Tanno, 2009; Watkins et al., 2008). When reflection is therapeutically guided, it might predate better metacognitive insight into one’s troubles and turn out as an indicator for recovery and reinstatement of meaning (Lysaker & Klion, 2017).

### Clinical Implications

Whereas experiencing meaning in life was predictive of a lower symptom burden, reflection about meaning in life was associated with more psychopathological symptoms cross-sectionally and, to a limited extent, longitudinally. Moreover, reflection about meaning in life was not significantly associated with future experiencing of meaning (see Table 2 and Anoschin et al., 2024S). We tentatively conclude that therapeutic approaches seem promising which encourage individuals to explore new contexts where meaning can be intuitively experienced (Hirsh, 2013; Shin & Steger, 2014). This suggestion is in line with research highlighting the importance of experiential appreciation for meaning in life (Kim et al., 2022).

One mechanism under discussion through which meaning in life exerts beneficial effects (e.g., Dulaney et al., 2018) is the stress buffer hypothesis (He et al., 2023). For instance, Eisenbeck et al. (2022) found that meaning-centered coping strategies were the best predictors of lower psychological distress and greater well-being during the COVID-19 pandemic. Importantly, the authors went beyond mere cognitive aspects when defining meaning-centered coping. They explicitly included behavioral and emotional manifestations of meaning in life, such as life appreciation, pro-sociality and engagement in meaningful activities. Therefore, interventions that promote meaningful action may prove effective in the treatment of depression and personality pathology (Eakman, 2014; Van Tongeren et al., 2016). Despite being recognized as an important factor for understanding the dynamics of psychopathology (Steger, 2022), little attention has been paid to the systematic investigation of meaning in life in psychotherapeutic settings. Future research should establish how meaning is co-created within a therapeutic relationship (Summers, 2001) and how patients may benefit from meaning-centered interventions (e.g., Böhmer et al., 2022; Breitbart et al., 2015).

Regarding reflection about meaning, it must be highlighted that reflection is not synonymous with comprehension. Hence, reflection could be misguided in certain contexts, for instance, when it is ruminative (Watkins & Roberts, 2020). An affective component is strongly implied in the subjective experience of meaning (Hicks et al., 2010). Therefore, to experience meaning, it might not suffice to abstractly reflect about it.

### Limitations

The generalizability of our results to the clinical context is limited because we drew our sample from the general population. Notably, an unusually large proportion of our sample reported clinically relevant levels of psychopathology, which could be attributable to greater psychological distress observed during the COVID-19 pandemic (Daly & Robinson, 2022) or to peculiarities of the online participant pool (Ophir et al., 2020). In addition, participants were not actively reminded to take part in the follow-up assessment, and this procedure may have introduced self-selection bias. Furthermore, only two assessment time-points were employed, limiting conclusions about temporal dynamics between the investigated constructs. Future research should explore in more temporal detail the covariation of experienced meaning, reflection on meaning and psychopathology. For example, ecological momentary assessments (Kaurin et al., 2023; Steger & Kashdan, 2013) may provide much needed empirical insights about within-person mechanisms on a timescale of days or even hours. Our conclusions may be further limited by the moderate reliability of the utilized “experience of meaning in life” subscale. Future studies could consider longer scales that tend to reach higher internal consistency. Additionally, experimental designs should be employed to test causal hypotheses formulated on the basis of the present results. For example, one could induce reflection on meaning in life and compare the effects on measures of affect and well-being between healthy participants and those undergoing treatment.

## Conclusion

The present findings imply that the experience of meaning in life could aid in the reduction of depressive symptoms, and possibly personality functioning impairments over time. Conversely, psychopathological symptoms may reduce the experience of meaning in the future, as indicated by exploratory cross-lagged analyses. Cross-sectionally, a greater depressive burden is accompanied by increased reflection about meaning in life. Our data tentatively suggests that reflection is also longitudinally associated with depressive symptoms and personality functioning impairments. However, further studies are needed to conclude whether reflection may be causally linked to an exacerbation of psychopathological symptoms. Scholars and practitioners may be well advised to consider the role of both reflective and experiential components of meaning in life for symptom change.

## Supplementary Materials

The Supplementary Materials contain the following items:

- The preregistration for the study (Remmers et al., 2022S)
- The raw data supporting the conclusions of the article (Remmers et al., 2024S)
- Additional information: The supplementary file includes descriptive statistics and regression tables for the full sample of *n* = 538 participants. Additionally, it includes exploratory SEMs testing longitudinal bi-directional associations between meaning in life and psychopathology measures (Anoschin et al., 2024S).

AnoschinA.
ZürnM. K.
RemmersC.
 (2024S). Supplementary materials to "Longitudinal associations of experiential and reflective dimensions of meaning in life with psychopathological symptoms"
[Additional information]. PsychOpen. 10.23668/psycharchives.15036
PMC1163673939678316

RemmersC.
ZimmermannJ.
TopolinskiS.
ZürnM. K.
 (2022S). Intuition and meaning in life in persons with varying level of depressive symptoms and impairments in personality functioning: A follow-up study
[Preregistration]. PsychOpen. https://osf.io/3zprc
10.1002/jclp.2348736693351

RemmersC.
ZürnM. K.
TopolinskiS.
ZimmermannJ.
AnoschinA.
 (2024S). Intuition and meaning in life in persons with varying level of depressive symptoms and impairments in personality functioning
[Research data]. PsychOpen. https://osf.io/8fx9s
10.1002/jclp.2348736693351

## Data Availability

The raw data supporting the conclusions of this article is available in an online repository on the Open Science Framework (OSF) (see Remmers et al., 2024S).

## Reporting Guidelines

In writing the manuscript, we followed the JARS-Quant reporting standards for studies using no experimental manipulation.
